# Publisher Correction: Measuring the ionisation fraction in a jet from a massive protostar

**DOI:** 10.1038/s41467-019-12105-9

**Published:** 2019-09-03

**Authors:** R. Fedriani, A. Caratti o Garatti, S. J. D. Purser, A. Sanna, J. C. Tan, R. Garcia-Lopez, T. P. Ray, D. Coffey, B. Stecklum, M. Hoare

**Affiliations:** 1 Dublin Institute for Advanced Studies, School of Cosmic Physics, Astronomy & Astrophysics Section, 31 Fitzwilliam Place, Dublin, D02 XF86 Ireland; 2 School of Physics, University College Dublin, Belfield, Dublin, 4 Ireland; 3 Max-Planck-Institut für Radioastronomie, Auf dem Hügel 69, 53121 Bonn, Germany; 4 Department of Space, Earth & Environment, Chalmers University of Technology, SE-412 93 Gothenburg, Sweden; 5 Department of Astronomy, University of Virginia, 530 McCormick Road, Charlottesville, VA 22904-4325 USA; 6 Thüringer Landessternwarte Tautenburg, Sternwarte 5, 07778 Tautenburg, Germany; 7 School of Physics and Astronomy, University of Leeds, Leeds, LS2 9JT UK

**Keywords:** Interstellar medium, Stars, Stellar evolution, Astronomy and astrophysics

Correction to: *Nature Communications* 10.1038/s41467-019-11595-x, published online 9 August 2019.

The original version of this Article contained an error in last sentence of the legend to Table 4, which incorrectly read ‘^a^Assuming a tangential velocity of 150–300 km^−1^ from the proper motions of ref. ^22^’ The correct version states ‘150–300 km s^−1^’ in place of ‘150–300 km^−1^’.

The original version also contained errors in Table 5, in which the headings of the fifth, sixth and seventh rows incorrectly read, ‘$\dot M_{_{{\mathrm{ionised}}}}$ (×10^−6^ *M*_⊙_ y^−1^)’, ‘$\dot M_{{\mathrm{ejec}}}$ (×10^−5^ *M*_⊙_ y^−1^)’ and ‘$\dot P_{_{{\mathrm{ionised}}}}$ (×10^−4^ *M*_⊙_ y^−1^ km^−1^)’, instead of the correct ‘$\dot M_{_{{\mathrm{ionised}}}}$ (×10^−6^ *M*_⊙_ yr^−1^)’, ‘$\dot M_{{\mathrm{ejec}}}$ (×10^−5^ *M*_⊙_ yr^−1^)’ and ‘$\dot P_{_{{\mathrm{ionised}}}}$ (×10^−4^ *M*_⊙_ yr^−1^ km s^−1^)’, respectively.

The last sentence of the legend to Table 5 originally incorrectly read ‘^a^Assuming a tangential velocity of 150–300 km^−1^ from the proper motions of ref. ^22^’ The correct version states ‘150–300 km s^−1^’ in place of ‘150–300 km^−1^’.

The original version also contained an error in the third sentence of the first paragraph of the ‘Ionised mass-loss rate on source’ section of the Methods, which incorrectly read ‘From our observations we obtain the following parameters for core B: *S*_5.8_ = 0.794 ± 0.03 mJy, *ν* = 5.8 GHz, *v*_*j*_ = 600 ± 100 km^−1^, θ_0_ = 52.3° ± 4.4°, *D* = 2.2 kpc, *T* = 10,000 K, the resulting ionised mass-loss rate is 1.81 ± 0.33 × 10^−6^ M_⊙_ yr^−1^, consistent with ref. ^22^.’ The correct version states ‘*v*_*j*_ = 600 ± 100 km s^−1^’ in place of ‘*v*_*j*_ = 600 ± 100 km^−1^’.

This has been corrected in both the PDF and HTML versions of the Article.

