# Supplementary material for: Measuring the ionisation fraction in a jet from a massive protostar
Source: Nat Commun. 2019 Aug 9;10:3630. doi: 10.1038/s41467-019-11595-x (PMC6689082; doi:10.1038/s41467-019-11595-x)
Supplement: Supplementary file 1 — Peer Review File [file 41467_2019_11595_MOESM1_ESM.pdf]

Editorial Note: This manuscript has been previously reviewed at another journal that is not operating a transparent peer review scheme. The manuscript was considered suitable for publication without further review at *Nature Communications.*
